# Social functioning as a domain of quality of life in adults with obesity: results of the phase 2 validation of the *Obesity Quality of Life Instrument*

**DOI:** 10.13075/ijomeh.1896.02820

**Published:** 2026

**Authors:** Monika Szkultecka-Dębek, Aleksandra Gradowska, Marta Bem, Mariola Drozd

**Affiliations:** 1 University of Social Sciences, Faculty of Applied Sciences, Warsaw, Poland; 2 SWPS University, Warsaw, Poland; 3 Qualitas Vitae Institute, Foundation, Warsaw, Poland; 4 Medical University of Lublin, Department of Humanities and Social Medicine, Lublin, Poland

**Keywords:** obesity, quality of life, social functioning, work environment, instrument, OQI-3

## Abstract

**Objectives:**

The aim of this study is to examine social functioning as a key domain of obesity-related quality of life (QoL) using the updated *Obesity Quality of Life Instrument* (OQI-3) v. 2.

**Material and Methods:**

The study was conducted in 2024 among adult patients diagnosed with obesity treated at a hospital dietary clinic in Warsaw, Poland. The study continued the validation work on the OQI-3 instrument following a pilot study. The updated OQI-3 v. 2 consists of 3 sections: 1) gender-adjusted workplace vignettes assessing perceived attitudes and emotions, 2) a multiple-choice list of obesity-affected daily activities, and 3) a 16-item Likert scale assessment of the impact of obesity on daily functioning. Descriptive analyses, item analysis, internal consistency reliability, and principal component analysis (PCA) were performed.

**Results:**

A total of 164 participants were included (125 women and 39 men), with BMI of mean ± standard deviation 40.91±7.68 kg/m², corresponding to class III obesity. The vignette-based section revealed predominantly negative workplace experiences, including discomfort, unfriendly behaviors, and reduced interest, accompanied mainly by emotions such as sadness, stress, frustration, and reduced self-worth. In the daily activities section, rapid fatigability was the most frequently reported difficulty, followed by problems with climbing stairs, poor physical condition, and clothing size issues. The third section demonstrated high internal consistency (Cronbach's α = 0.895). Principal component analysis identified 3 components – interpersonal functioning, self-care, and physical activity – explaining 58.55% of the total variance, each showing satisfactory to very good reliability (α = 0.714–0.877).

**Conclusions:**

*Obesity Quality of Life Instrument* v. 2 may be a reliable and psychometrically solid tool for assessing obesity-related quality of life, with particular strength in capturing impairments in social and interpersonal functioning. The findings highlight the substantial impact of obesity on workplace experiences, daily activities, and social relationships, supporting the relevance of social functioning as a critical QoL domain in adults with obesity.

## Highlights

- *Obesity Quality of Life Instrument* (OQI-3) v. 2 demonstrated high reliability and strong psychometric properties.
- Social and interpersonal functioning emerged as a key impaired quality of life domain.
- Workplace-related stigma and negative emotions were frequently reported.
- Physical activity limitations showed the greatest obesity-related difficulties.

## INTRODUCTION

Obesity continues to pose a significant burden to global health. According to epidemiology data the number of patients suffering from this disease is increasing and it affects not only adult population but more and more the youngest population as well. World Health Organization data from 2022 show 2.5 billion adults being overweight, and according to 2024 data 35 million children <5 years old were overweight [1].

Based on the WHO definition in case of adult population, overweight is a BMI ≥25 kg/m^2^, and obesity corresponds to a BMI ≥30 kg/m^2^ [1].

The health related consequences of the disease are broad and patients are at risk of developing numerous diseases such as type 2 diabetes, cardiovascular diseases or certain cancers, leading to increased mortality [2,3]. Despite its well-documented impact on physical health, obesity has also been consistently associated with significant impairments in health-related quality of life (HRQoL), affecting physical, emotional, and social functioning. Patients with obesity often report limitations in daily activities, psychological distress, and reduced social functioning. Reduced mobility, greater number of obesity related comorbidities, are among other factors influencing physical domains of patient's quality of life (QoL). Nevertheless, obesity is closely related with psychological distress, including lower self-esteem and anxiety or depression. Importantly, weight-related stigma and negative social experiences may also contribute to limited social life, negatively affect interpersonal relationships, and result in poorer social functioning [4–10]. Several validated tools have been developed to assess obesity-related QoL, including the *Impact of Weight on Quality of Life-Lite*, or the *Laval Questionnaire*. These instruments assess multiple aspects of QoL affected by obesity, including physical functioning, emotional well-being, and social aspects of daily life. A more detailed overview of existing tools and their characteristic has been presented in the previous manuscript which described the results of the *Obesity Quality of Life Instrument* (OQI-3) pilot study [11]. However, despite a number of existing tools measuring health related QoL there are areas such as the impact of obesity on patients' daily activities or the social functioning which has not been explored enough so far. In order to address the identified gaps, a research was conducted introducing a new tool OQI-3 to assess patients' social functioning drawing special attention to daily functioning and work related activities. The pilot study has proven the internal consistence of the OQI-3 tool [11]. However, also i ndicated the need for further validation in a larger sample and minor modifications to the questionnaire structure. Therefore, the aim of this study is to examine the social functioning as a significant QoL domain affected by the disease by using the updated version (v. 2) of the OQI-3 tool among adult population with obesity.

## MATERIAL AND METHODS

### Study design and procedure

The study was performed as a continuation of the research focused on OQI-3, which is a new tool specific for obesity disease and its impact on patients QoL [11]. The initial results from the pilot study have proven the internal consistency of the first version of OQI-3. However, validation of the tool required testing in a larger sample to confirm the pilot findings and provide more robust estimates of its psychometric properties. Based on the pilot study findings the questionnaire was modified by clarifying questions, providing multiple choice answers options to first and second section, and by adding items to the third section. As the study involved a non-interventional, questionnaire-based survey, formal ethical approval was not required under current Polish regulations. However, as the study was conducted as a continuation of a pilot study that had received approval from the Ethics Committee of the University of Social Sciences in Warsaw, Poland (No. 3/24), the study was notified to the Ethics Committee. The procedure was conducted following the Hospital Director's recommendation, and all participants provided written informed consent before participation. The data collection was performed between June–December 2024, among patients of the hospital dietary clinic in Warsaw, Poland. Inclusion criteria were age 18–65 years, current treatment for obesity, and provision of written informed consent to participate. Participants were informed about the purpose of the study, data anonymity, the voluntary nature of participation, and their right to withdraw consent and discontinue the questionnaire at any time. Patients were approached in person during their visit to the hospital's dietary clinic and asked to complete the questionnaire. Those who consented completed the OQI-3 v. 2 questionnaire independently, either while waiting for their appointment or immediately afterward.

### Research tool

#### Conceptual framework

The development of the OQI-3 v. 2 instrument was based on the contemporary HRQoL frameworks that conceptualize QoL as a multidimensional construct reflecting not only physical health, but also daily functioning, social participation, and social environment influences. The conceptual foundations of the questionnaire were based on 3 complementary approaches: the Wilson and Cleary model, the World Health Organization Quality of Life (WHOQOL) framework, and the International Classification of Functioning (ICF). Each of these contributes a slightly different perspective – and it is precisely their integration that formed the basis of the developed instrument. The Wilson and Cleary model conceptualizes QoL as a construct linking objective clinical aspects (such as symptoms and health status) with patients' actual functioning and their subjective day-to-day experiences. The WHOQOL framework broadens this perspective by emphasizing that QoL extends beyond physical health to include psychological well-being, social relationships, and environmental context. In turn, the ICF is particularly useful where QoL is understood in terms of everyday functioning – specifically, the extent to which an individual is able to participate in social, occupational, and family life. These 3 approaches were not treated as rigid frameworks to be reproduced, but rather as a starting point for determining which domains are truly relevant in the context of adults with obesity. The questionnaire itself is not a direct operationalization of any single model into a set of items. Instead, emphasis was placed on capturing the shared elements across these approaches – particularly in the domains of social functioning, interpersonal experiences, and limitations in daily life, which, in clinical practice, often prove to be of central importance. Within these frameworks, obesity is conceptualized not only as a medical condition, but as a chronic health state that generates persistent limitations in activity and participation exposing individuals to adverse environmental responses, including stigma and discrimination. Therefore, the OQI-3 v. 2 was designed as a functioning-oriented and participation-oriented instrument, focusing on how obesity affects everyday life, social relationships, and occupational functioning [1,3,9,12–23].

The OQI-3 v. 2 tool consists of 3 sections, where the first section is a gender adjusted vignette, describing a set of 4 work environment related scenarios. The use of vignette-based methodology allows for the assessment of situational and context-dependent aspects of QoL, reducing social desirability bias and enabling respondents to project their experiences onto standardized scenarios [24,25]. The scenarios include searching for a job situation, relations with peers at work and relations with superiors. The aim of the vignette is to gather qualitative information on observed attitudes and respondents' emotions in each of the described situations. Based on the OQI-3 pilot study results and respondents' feedback indicating difficulty in naming attitudes and emotions for each scenario, the second version of the OQI-3 tool was modified. The situational descriptions were followed by a list of different attitudes and emotions from which the respondent could chose those they considered applicable. These options derive from participants' responses in the pilot study, in which open-ended questions were used in this section of the tool. In this first section of the instrument, the first scenario included 11 observed attitudes and 19 experienced emotions, whereas the subsequent scenarios included 20 observed attitudes and 29 emotions, respectively.

Responses obtained from the vignette section were analyzed descriptively (i.e., frequency distributions of selected attitudes and emotions) and were not included in the psychometric validation procedures. This section was designed to provide contextual insight into participants' experiences rather than to assess the internal structure of the scale. The vignette data were not included in the core psychometric validation procedures (e.g., factor analy sis, reliability estimation), as their purpose was not to assess the internal structure of the scale but rather to enrich the interpretation of the findings.

The second section of the OQI-3 tool consisted of an openended question related to daily activities. Each study participant was asked to identify and provide information on the mostly affected daily activities, causing more difficulties for the respondent to perform them due to obesity. The OQI-3 v. 2 was modified by replacing the open-ended question with the option of multiple choice from a provided list of 25 activities. The list was created based on the answers obtained from respondents during the pilot phase of the OQI-3 questionnaire.

In both sections of the questionnaire (the first, covering all scenarios, and the second one), patients were also given the opportunity to provide an additional optional response in an “other” category, which was not included in the item numbering described above.

The third section of the OQI-3 v. 2 tool was expanded by additional 5 items compared to OQI-3, resulting in a total of 16 items representing different activities. The list of the activities was provided in a form of a table, and respondents were asked to rate each activity in terms of the impact of obesity on it. This section is based on a Likert scale, allowing for the following answers: high impact, medium impact, low impact, no impact, neutral. In the Likert scale, respondents were allowed to choose the answer that best reflected their experiences. The scale assessed the impact of obesity on a range of daily activities, ranging from very high to no impact. Additionally, a neutral option was included, indicating that the activity is perceived as irrelevant by the respondent, or that the respondent is unable to determine whether it has any impact or not.

### Statistical analysis

A minimum sample size of 150 participants was planned for this phase of the study. Statistical analyses were conducted using IBM SPSS Statistics, v. 29 (IBM Corp., Armonk, USA). Internal consistency reliability of the third section of the OQI-3 v. 2 was assessed using Cronbach's α. Prior to conducting PCA, sampling adequacy was assessed using the Kaiser-Meyer-Olkin (KMO) measure and Bartlett's test of sphericity; detailed results of these analyses are presented in the Results section, as they reflect data-specific diagnostics interpreted in the context of the obtained empirical findings.

Principal component analysis with Varimax rotation was conducted on the 16 items to obtain a clear and interpretable component structure, assuming low correlations between extracted components. The number of components was guided by eigenvalues >1. Dimensional structure was examined using principal component analysis, as described in the Results section.

## RESULTS

### Characteristics of the study population

A total number of 164 respondents participated in the study. The general characteristics of the studied population is presented in Table 1. Among women (N = 125), those aged 41–50 years constituted the largest group (30.4%, N = 38). The next age groups were 31–40 years and 51–60 years (22.4%, N = 28, in each category). Within the men group (N = 39), majority were aged 41–50 years and 51–60 years (28.2%, N = 11, in each group). The study group was dominated by respondents with higher and secondary education (48.8% and 42.7%, respectively) and living in a city (82.3%).

**Table 1:** Characteristics of the studied group of adult patients with obesity, hospital dietary clinic in Warsaw, Poland, June–December 2024

Variable	Participants (N = 164)	
	n	%
Gender		
female	125	76.2
male	39	23.8
Age^a^		
20–30 years	19	11.6
31–40 years	36	22.0
41–50 years	49	29.9
51–60 years	40	23.8
>60 years	19	11.6
Place of residence		
village	23	14.0
city	135	82.3
missing data	6	3.7
Education level		
primary	14	8.5
high school	70	42.7
university	80	48.8

a Total N = 163; 1 missing response.

Regarding the body parameters presented in Table 2, 4 variables were analyzed: height, weight, waist circumference, and BMI. For the first 3 variables, the sample size was N = 164, while for waist circumference, data were available for only 39 individuals, due to missing information in patient declarations. In this subsample, the waist circumference was mean (M) ± standard deviation (SD) 125.92±16.36 cm, and the median (Me) was 124.00 cm. The body weight in the sample was M±SD 117.21±27.45 kg, with Me = 111 kg and the BMI value in the entire sample was M±SD 40.91±7.68 kg/m², with Me = 39.61 kg/m². The obtained values indicate that the study group was characterized by, on average, class III obesity according to the WHO classification.

**Table 2:** Body parameters analysis based on data from adult patients with obesity, hospital dietary clinic in Warsaw, Poland, June–December 2024

Variable	Participants (N = 164)		
	M	Me	SD
Heigh [m]	1.688	1.680	0.095
Weight [kg]	117.20	111.00	27.453
BMI [kg/m^2^]	40.906	39.614	7.683

The distribution of BMI categories in the study sample is presented at Table 3 and it indicates that the largest proportion of participants were classified as class III obesity (BMI ≥ 40 kg/m²), accounting for 48.2% of the sample. Participants with class II obesity (BMI 35.0–39.9 kg/m²) constituted 28%, while 20.7% were classified as class I obesity (BMI 30.0–34.9 kg/m²). Overall, the sample was dominated by individuals with severe obesity (class II and III), representing >75% of participants, which is consistent with the high mean BMI observed in the study. At the same time, the distribution confirms heterogeneity within the sample, supporting the need to interpret BMI at the individual level rather than relying solely on the M value. A small proportion of participants (3%, N = 5) had a BMI <30 kg/m² and were not excluded from the analyses. This decision was based on the fact that the study was conducted in a clinical setting, and participant inclusion was determined by treatment status for obesity rather than a single BMI measurement. Those individuals had previously met the criteria for obesity and were in the process of weight reduction. Given the small proportion of such cases, their impact on the overall results was considered negligible, and their inclusion allowed for greater ecological validity of the study.

**Table 3:** Distribution of body mass index (BMI) categories in the study sample of adult patients with obesity, hospital dietary clinic in Warsaw, Poland, June–December 2024

Variable	Participants (N = 164)			Min.–max
	n	%	cumulative %	
BMI				
<30.0	5	3.0	3.0	24.91–29.30
30.0–34.9 (class I obesity)	34	20.7	23.7	30.08–34.96
35.0–39.9 (class II obesity)	46	28.1	51.8	35.01–39.89
≥40.0 (class III obesity)	79	48.2	100.0	40.06–65.54

BMI categories were defined according to the WHO classification.

The body parameters were also analyzed by gender. The height within women group was M±SD 1.65±0.06 m, with Me = 1.65 m, the body weight – M±SD 108.28±19.90 kg, with Me = 105 kg, and BMI – M±SD 39.57±6.70 kg/m², with Me = 38.49 kg/m². In the group of men, the height was M±SD 1.80±0.10 m, with Me = 1.80 m. The body weight was M±SD 145.82±28.95 kg, with Me = 144 kg and the BMI equaled M±SD 45.18±9.05 kg/m², with Me = 44.61 kg/m².

### OQI-3 v. 2 results

#### Part 1: Vignettes related to respondents' work environment – qualitative results

**Figure 1.**
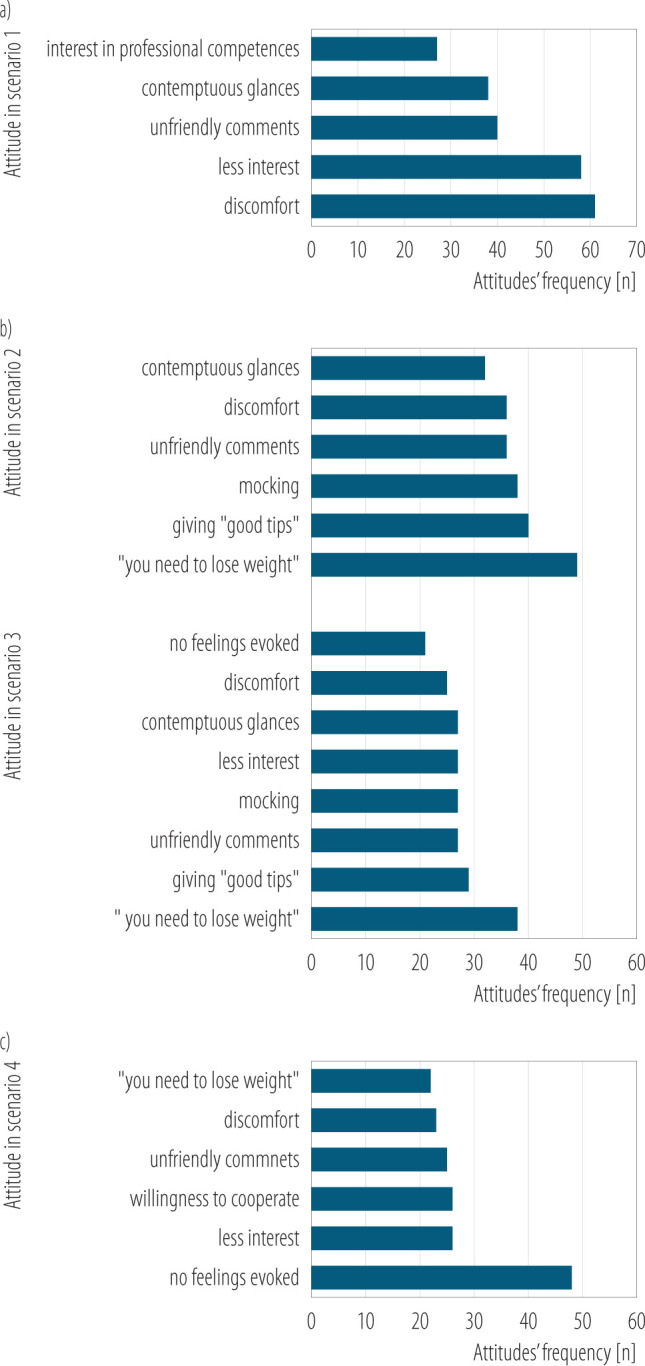
The most frequent attitudes from: a) scenario 1 (searching for a job), b) scenarios 2 and 3 (relationships with workplace peers), and c) scenario 4 (relationships with superiors) in 164 adult patients with obesity, hospital dietary clinic in Warsaw, Poland, June–December 2024

The most frequently observed attitudes by the respondents in the first scenario (searching for a job) were rather perceived as negative, as feeling discomfort (N = 61), less interest in the person (N = 58), unfriendly comments (N = 40), contemptuous glances (N = 38), however some of the respondents also declared the appearance of questions about professional competences (N = 27). The less frequently observed attitude was openness (N = 7). Regarding the scenarios focused on the relationships at work with peers (by gender) the most frequently declared attitudes were pointing out that “you need to lose weight,” giving “good tips,” mocking, unfriendly comments, feeling discomfort, contemptuous glances and less respondents stated that “no feelings evoked.” However, the attitudes observed in relations with superiors were mostly “no feelings evoked,” followed by the same frequency regarding willingness to cooperate, less interest in the person, unfriendly comments, feeling discomfort and pointing out that “you need to lose weight.” Figure 1 shows the most frequent attitudes for all scenarios.

Regarding emotions declared by the respondents and regarding the scenario 1, the most frequently reported emotional responses were stress (N = 79), feelings of reduced self-worth (N = 65), and frustration (N = 56). These were followed by helplessness, irritability and sadness. While the least commonly endorsed emotional response was a sense of being lost, declared by only 2 respondents.

In scenario 2, referring to relationships with peers, the most frequently reported emotional response was sadness (N = 68). This was followed by stress (N = 48), feelings of reduced self-worth (N = 46), and frustration (N = 38). Less frequently reported, but still notable, were calmness (N = 23) and withdrawal (N = 23). The least frequently reported emotional responses were feelings of rejection and a sense of injustice, each selected by 1 participant.

In scenario 3, the mostly reported emotional responses in relation to peers relationships were sadness (N = 67), stress (N = 55) and frustration (N = 33). Irritability and calmness were reported with equal frequency (N = 27 each). Less frequently endorsed responses, also still relatively common, included neutral feelings and perceived discrimination.

In Scenario 4, which concerned interactions with superiors in the workplace, sadness was the most commonly reported emotion (N = 44), followed by stress and calmness (N = 34 each) and neutral feelings (N = 33). Positive emotions and frustration were also commonly reported.

**Figure 2.**
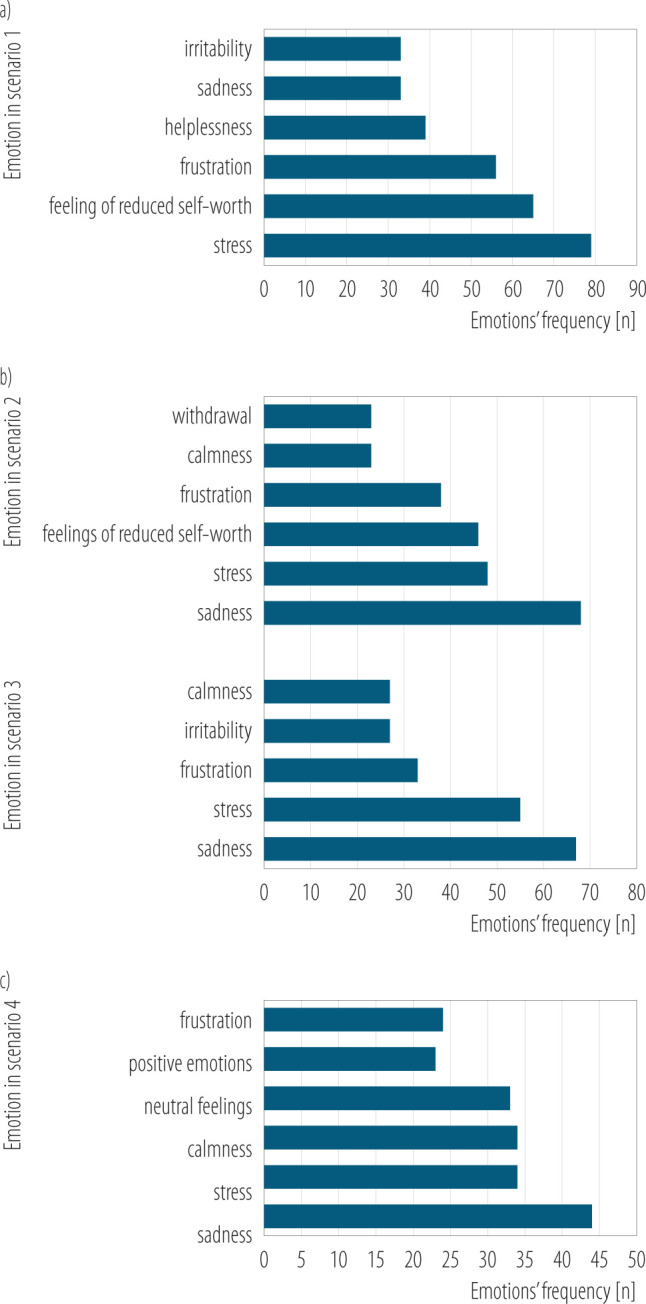
The most frequent emotions from: a) scenario 1 (searching for a job), b) scenarios 2 and 3 (relationships with workplace peers), and c) scenario 4 (relationships with superiors) in 164 adult patients with obesity, hospital dietary clinic in Warsaw, Poland, June–December 2024

The details related to the most frequently named emotions are presented at Figure 2.

#### Part 2: Impact of obesity on daily activities – qualitative results

**Figure 3.**
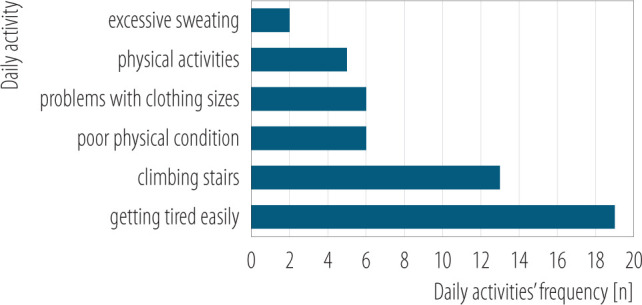
The most frequently affected daily activities (part 2 results) in 164 adult patients with obesity, hospital dietary clinic in Warsaw, Poland, June–December 2024

In the distribution of responses (Figure 3), item 1 (“getting tired easily”) clearly dominated. It is the most frequently selected category across the entire dataset, accounting for nearly 1 in 10 responses. High frequencies were also observed for item 4 (“climbing stairs”), item 15 (“poor physical condition”), and item 21 (“problems with clothing sizes”).

#### Part 3: Impact of obesity on daily activities – quantitative results

The reliability analysis of the third section of the OQI-3 v. 2 was conducted following a recoding of the Likert scale (originally: 1→3, 2→1, 3→2, 4→4, 5→5). This procedure was necessary to ensure that lower scores reflect low trait intensity and the neutral response (score 3) represents the conceptual midpoint of the scale (1 – no impact and 5 – high impact).

This transformation preserved the original ordinal structure of perceived burden while significantly improving the interpretability and content validity of the instrument. Importantly, sensitivity analyses conducted using the original coding yielded comparable reliability coefficients, demonstrating that the recoding procedure did not artificially inflate psychometric parameters [26–28].

The calculated Cronbach's α for the entire scale was α = 0.895, indicating high internal consistency. This indicates that the 16 items comprising the scale consistently measure the same psychological construct. The α value based on the standardized items was identical (α = 0.895), further confirming the stability of reliability regardless of differences in inter-item variance.

The next analysis step was to look into the difficulty of the items. The response format was 5 – high impact, 1 – no impact, high average – high level of difficulty/stronger impact of the problem, low average – low level of difficulty/weaker impact of the problem (the higher the average, the greater the difficulty in a given area). Normalized mean score (0–1): 0 – minimal difficulty, 1 – very great difficulty. The greatest difficulties (p > 0.70), were reported by respondents in the following areas: increasing physical activity (p = 0.848), practicing sports (p = 0.777), and worth mentioning health care (p = 0.652). The category referred to moderate difficulties (p = 0.40–0.60) included the majority of items: housework, social life, relationships with partner, perception at work, sexual activity, most areas of self-care. These are areas of functioning where problems are noticeable and subjectively felt, but not extreme. These items may have the best discriminatory power on the scale. Least difficulties (p < 0.30) were: relationships with family (p = 0.246) and relationships with friends (p = 0.258).

### Item correlation matrix analysis

The analysis of the inter-item correlation matrix revealed that the vast majority of items exhibited moderate interrelationships, with coefficients ranging 0.20–0.50 and an inter-item correlation of M = 0.35. These values indicate adequate linkage to the construct and a lack of content redundancy, falling within the recommended range of 0.15–0.50 for scale homogeneity [26,29]. Importantly, no items exhibited very low correlations (<0.10), suggesting that all items contribute meaningfully to the construct and no “statistical outliers” were present.

The only pair that exceeded the commonly accepted threshold suggesting potential content duplication were the items “contacts with partner” and “sexual contacts” (r = 0.737). Such a high correlation indicates that these items measure a very similar aspect of functioning and may be considered for combination or refinement in subsequent validation stages. The remaining high correlations (range 0.60–0.69) concerned social items (e.g., establishing contacts and social life), which is consistent with the construct logic and does not exceed the criteria for redundancy in the psychometric literature.

Analysis of item statistics revealed that most items had good psychometric properties, achieving item-total correlations >0.50 and acceptable or high measure of sampling adequacy (MSA) values (Table 4). Analysis of item statistics showed that most items had good psychometric properties, achieving high sampling adequacy for all 16 items of the OQI-3 v. 2 scale. Individual MSA coefficients for all variables exceeded the threshold of 0.70, reaching values 0.752–0.921. These results, interpreted in accordance with Kaiser's standards, confirm the very good structure of the tool and justify the inclusion of all questions in further psychometric analyses. Item-total correlations were >0.50 and obtained acceptable or high MSA values [26,29,30].

**Table 4:** Total item statistics results of the *Obesity Impact on Functioning in Society Questionnaire* (OQI-3 v. 2), hospital dietary clinic in Warsaw, Poland, June–December 2024

OQI-3 v. 2 item	Scale M^a^	Scale variance^a^	Item correlation^b^	Cronbach's α^a^	MSA
Shopping	43.29	177.36	0.335	0.897	0.770
Housework	42.70	170.78	0.487	0.892	0.852
Contacts with others	43.15	165.86	0.588	0.888	0.852
Free time	43.16	169.15	0.572	0.889	0.901
Social life	43.01	162.49	0.724	0.883	0.837
Relationship with partner	43.03	162.16	0.681	0.884	0.832
Relationship with family	43.80	170.95	0.553	0.889	0.756
Relationship with friends	43.75	169.56	0.591	0.888	0.795
Practicing sports	41.68	170.96	0.509	0.891	0.769
Physical activity	41.39	176.24	0.450	0.893	0.752
Perception in the work environment	42.99	169.55	0.526	0.890	0.916
Whole body hygiene	43.27	167.47	0.569	0.889	0.850
Health care	42.18	167.06	0.551	0.889	0.863
Hair care	43.25	169.34	0.493	0.892	0.844
Sexual activity	42.81	163.57	0.636	0.886	0.844
Meals preparation	43.34	163.96	0.636	0.886	0.921

MSA – measure of sampling adequacy.

a After item removal.

b Total.

The strongest items concerned areas of social functioning, such as “social life,” “relations with partner,” “sexual activity,” and “contacts with others.” The only item with significantly weaker parameters was “shopping,” with an item-total correlation of 0.335 and a squared multiple correlation of only 0.257, suggesting a weaker relationship with other scale items. The remaining items had adequate parameters, and their removal would not have led to an improvement in Cronbach's α, confirming their adequate contribution to the scale's construction.

Based on Kaiser's recommendations and current psychometric standards, the adequacy of the data for components analysis was assessed [31]. The KMO test result of 0.835, indicates a very good sample quality and confirms that the variables shared sufficient common variance to enable the extraction of stable components. Additionally, Bartlett's test of sphericity was statistically significant (χ²(120) = 941.749, p < 0.001), indicating that the correlation matrix differed significantly from the unique matrix. The obtained results clearly support the validity of principal component analysis for the studied scale.

### Principal components analysis

Principal components analysis (PCA) using the Kaiser's criterion identified 3 components with eigenvalues >1. These components explained a total of 58.55% of the variance, a satisfactory level consistent with typical psychometric standards. Before rotation, the first dimension explained 39.53% of the variance, however after Varimax rotation, the variance distribution became more even – the first component explained 27.56% of the variance, the second 15.86%, and the third 15.14%. This indicates that rotation increased the clarity of the component structure, ensuring that each component better represents a coherent set of items. The analysis identified 3 clear dimensions describing the participants' daily functioning (Table 5). Varimax rotation was applied to obtain a clearer and more interpretable component structure. The first component grouped social activities – contacts with partners, family, and friends, social life, and establishing relationships – and can be interpreted as an indicator of interpersonal functioning. The second component reflected behaviors related to self-care, such as personal hygiene, hair care, health care. The third component encompassed physical activities, primarily sports and increasing physical activity, and housework, which allows it to be interpreted as a dimension of physical activity. The component structure is clear, and high component loadings indicate a good fit between the items and the identified dimensions.

**Table 5:** Rotated components matrix of the *Obesity Impact on Functioning in Society Questionnaire* (OQI-3 v. 2), hospital dietary clinic in Warsaw, Poland, June–December 2024

OQI-3 v. 2 item	Dimension		
	component 1	component 2	component 3
Social life	0.803		
Relations with partner	0.795		
Relations with friends	0.769	0.319	
Relations with family	0.726		
Contacts with others	0.705		
Sexual activity	0.659		
Free time	0.579		0.386
Meals preparation		0.442	
Perception in the work environment	0.408		
Whole body hygiene		0.757	
Hair care		0.733	
Health care		0.649	
Housework			0.521
Shopping		0.429	0.321
Practicing sports			0.848
Physical activity			0.828

Component 1 – social engagement; component 2 – self-care and activities of daily living; component 3 – physical activity.

Component 1 Cronbach's α reliability was 0.877, indicating very good internal consistency of the scale. A similar α value based on standardized items (0.876) confirms that the items have comparable variance and the scale structure is stable. The 8-item scale is characterized by high reliability and can be considered a reliable measurement tool.

The analysis of component 2, the 4-item scale, resulted in Cronbach's α coefficient of 0.767, indicating satisfactory reliability. A similar α value based on standardized items (0.768) confirms that all items have similar variance and interact well with each other. These items accurately reflect a single common construct – self-care and daily health and hygiene – while maintaining content differentiation. All scale items demonstrate moderate to strong positive correlations (r = 0.373–0.553), confirming their interconnectedness and shared embeddedness in the self-care construct. The strongest correlation concerns whole-body hygiene and health care (r = 0.553), indicating the centrality of these items. The other correlations remain within the moderate range, demonstrating the scale's coherence and lack of content redundancy. These results suggest that the scale has sufficient internal consistency, especially considering its short nature.

The obtained value of Cronbach's α = 0.714 for component 3 indicates satisfactory internal consistency of the scale, in line with accepted standards for short, 4-item subscales. The similar α value based on standardized items (0.719) confirms that the items are characterized by comparable variance and a uniform measurement structure. These results indicate acceptable subscale reliability.

The analyses focused exclusively on the third section of the questionnaire, while data from the vignette section were analyzed descriptively and are presented as qualitative contextual findings.

## DISCUSSION

In the following discussion, the authors interpret the main findings of the study while situating them within the broader literature and psychometric framework.

According to the analysis, women clearly outnumbered men in the study, which is a factor to be taken into account when interpreting the results and drawing conclusions about the entire population. However, it is worth noting that in the pilot phase, the gender distribution was similar (65.9% vs. 34.1%). The predominance of women in the present sample, while limiting generalizability, may also reflect gender-specific patterns of health-promoting behaviors and sensitivity to the social and emotional aspects of obesity. Previous research suggests that women may experience higher social evaluations related to body weight, which may partially explain the predominance of emotional and interpersonal themes observed in this study [20].

Significantly higher number of women participating in the study maybe due to the fact that women are more willing to participate in research or that they tend to seek specialist advice when dealing with weight problems more frequently than men.

Referring to the age structure of both women and men groups it indicates a predominance of individuals in middle age and late adulthood. Over 75% of women and nearly 80% of men were 31–60 years old, confirming the mature nature of the study sample.

The vast majority of the study sample were living in a city (82.3%), indicating a clear overrepresentation of city residents, which may have interpretive significance with respect to the analyzed variables, especially if they are related to access to services, lifestyle, or socioeconomic status. On the other hand, a similar distribution was observed in the OQI-3 pilot study, where city residents constituted 82.9% of the study population [11]. It is worth noting that patients treated at the hospital outpatient clinic did not have to be residents of Warsaw, but could be arriving from various locations in Poland. Similarly to the results obtained in the OQI-3 pilot study, in this phase of the study the structure of education indicates a high level of education of the studied sample: >90% of respondents had at least secondary education [26].

According to patients answers the problems related to physical activity and health are those identified as the most intense, which is consistent with the research on the functioning of people with obesity and chronic diseases where the areas of movement and health are the most often severely impaired elements of daily functioning. Such findings were confirmed in a systematic review analyzing the association between obesity, musculoskeletal health, and functional mobility among women where authors confirmed that higher BMI significantly impacted mobility-related limitations [32]. The research performed with OQI-3 v. 2 found that the items related to physical activity and health care may be more challenging for participants since they refer to behaviors requiring high levels of motivation, commitment, and resources, and are highly normative and susceptible to social evaluation [27]. This can generate greater response discomfort and greater individual variability. These constructs are also broad and subjectively interpreted, which can increase response variance. Respondents report relatively low difficulties in social relationships. These may be the areas least affected by difficulties, but protective mechanisms (e.g., avoiding disclosing problems in close relationships) may also be at work. A study focused on overweight women suppressing emotions to accommodate their male partners might illustrate such behaviors. The authors confirmed that women with a higher BMI reported high levels of suppressing emotions. By withholding negative affect to maintain relationship harmony and accommodate their partners, these women effectively reduce their partners' negative perceptions. However, they reported eating more than normal, which increases the risk of overeating [33]. Some interpretations should be viewed as cautious extrapolations or contextual hypotheses, supported by prior literature rather than directly by the authors' study data. The strong overlap observed between items referring to partner contact and sexual relations suggests that, in the studied population, intimate relationships are perceived as a unified aspect of social functioning rather than distinct domains. This finding may have implications for understanding how obesity-related stigma and body image concerns may simultaneously affect emotional closeness and sexual well-being.

Although respondents reported relatively lower levels of difficulty with regards to close social relationships, the social and interpersonal components demonstrated strong psychometric properties and constituted the strongest component in the scale. This suggests that social functioning, although not always perceived as severely impaired, represents a coherent and significant domain of obesity-related QoL, potentially characterized by subtle but persistent psychosocial burden. While physical limitations, such as limited capacity for physical activity, represented the most significant challenge, social dimension demonstrated strong discriminatory power and high reliability, emphasizing their importance in comprehensive QoL assessment.

The workplace emerged as a particularly sensitive context for obesity-related stigma, with respondents frequently reporting discomfort, unpleasant behaviors, and emotions such as sadness, stress, and low self-esteem. Other studies report similar findings regarding obesity related stigma, e.g., pointing out to the prevalence of weight discrimination in the USA which during the past decade has increased by 66%, and corresponds to the rates of racial discrimination, especially among women [20]. Previous research indicates that weight-based discrimination is one of the most prevalent forms of discrimination in Western societies and has increased by approx. 66% over the past decade, particularly among women [15].

The findings from the OQI-3 v. 2 study point to employment-related interactions as a distinct and significant source of psychosocial burden, highlighting the value of vignette-based assessments in capturing situational aspects of QoL that may be overlooked in traditional questionnaire formats [15].

The dimensions characterized by moderate difficulty, such as social life, relationships, and self-care, may be particularly clinically relevant. Such components often provide the greatest discriminatory power and sensitivity to change, making them valuable targets for monitoring patient-reported outcomes and assessing the effects of interventions over time.

The robustness of the multidimensional structure is further supported by high measures of sampling adequacy, with individual MSA values ranging 0.75–0.92. Particularly strong MSA values for items related to work and selfcare suggest that these components constitute central components of obesity-related QoL, reinforcing the conceptual validity of the identified subscales. The excellent internal consistency of the OQI-3 v. 2 (Cronbach's α = 0.895) indicates that the instrument meets psychometric standards for group-level research and may be useful as a structured clinical screening tool. Further studies should examine test–retest reliability and responsiveness to change. The inter-item correlation (M = 0.35), falling within the optimal range recommended for HRQoL instruments, suggests conceptual coherence without item redundancy, supporting the scale's precision in capturing diverse yet connected aspects of obesity related QoL. Importantly, these psychometric properties provide a solid preliminary basis for interpreting the observed patterns in physical, social, and occupational functioning, supporting the relevance and validity of the identified domains. Although 1 item related to shopping demonstrated a comparatively lower item–total correlation, its adequate sampling adequacy and contribution to content validity justify its inclusion. This supports the notion that daily functional challenges associated with obesity extend beyond health behaviors to encompass routine activities that are essential for independent living.

These results support the inclusion of a structured QoL assessment in routine care for individuals with obesity. The OQI-3 v. 2 can support clinicians in identifying psychosocial vulnerabilities, particularly in the social and occupational contexts, as well as to tailor multidisciplinary interventions that address both the physical and social dimensions of obesity.

### Study limitations

The study was conducted in a group of patients who are already undergoing treatment for obesity or who plan to initiate a treatment, i.e., are aware of their disease and the need for therapy as well as what could be the possible health consequences of no treatment.

This study has several limitations. First, the sample size study involved a modest clinical sample, however, it represents a clinical population actively participating in a structured obesity treatment program, which ensures its relevance for this stage of instrument validation. Second, women were overrepresented in the sample, which may limit generalizability. This predominance reflects gender-specific patterns of healthcare-seeking behavior and participation in research, as discussed earlier. Third, the study was conducted at a single center. However, patients originate from a variety of locations, supporting the relevance of the findings beyond a strictly local population. Finally, test–retest reliability, as well as convergent and divergent validity assessments, were not performed in this phase; these psychometric evaluations are planned for the subsequent phase of the study.

## CONCLUSIONS

The findings of this study suggest that the OQI-3 v. 2 may be a reliable and psychometrically solid tool for assessing obesity-related QoL in adults, with particular strength in capturing impairments in social and interpersonal functioning.

The findings highlight the substantial impact of obesity on workplace experiences, daily activities, and social relationships, supporting the relevance of social functioning as a critical QoL domain in adults with obesity.

The high internal consistency of the scale, clear component structure, and satisfactory reliability of the identified subscales support its potential usefulness both as a comprehensive QoL measure and as a tool for examining specific functional dimensions.

Importantly, the results highlight the key role of social and interpersonal functioning in obesity. Difficulties in workplace interactions, perceived negative attitudes from others, and emotionally demanding social situations were consistently reported, indicating that obesity related impairment may extend beyond physical limitations to include potential psychosocial consequences. The vignette-based section of the instrument appears to be particularly valuable in capturing emotional and attitudinal responses to socially relevant situations, especially in professional settings.

Furthermore, the identification of 3 distinct dimensions – interpersonal functioning, self-care, and physical activity – highlights the multidimensional nature of obesity-related QoL.

Taken together, these results support the inclusion of social functioning as a key domain in both clinical assessment and intervention planning for adults with obesity. The OQI-3 v. 2 could serve as a valuable tool in clinical practice and research, enabling healthcare professionals to identify areas of psychosocial vulnerability, monitor patient-reported outcomes, and tailor interventions. The tool could be integrated into multidisciplinary teams (dietitians, psychologists, surgeons) as a routine screening tool for patients with obesity.

Future studies should assess convergent and discriminant validity to support its use in individual clinical assessment. Finally, the assumed psychometric structure will be formally tested using confirmatory analysis to evaluate the model fit to the hypothesized factor structure.

Further research should focus on evaluating the responsiveness of OQI-3 v. 2 to clinical change, examining gender-specific response patterns, and validating the instrument in socioeconomically and geographically diverse populations. In particular, additional validation in male populations would be beneficial to ensure the tool's stability across genders.
